# Case report: Levodopa challenge test is important in identifying dopamine-induced freezing of gait in patient with Parkinson’s disease

**DOI:** 10.3389/fnhum.2024.1464152

**Published:** 2024-09-04

**Authors:** Shan-Shan Xu, Shi-Guo Zhu, Cheng-Xiang Yuan, Guo-Ling Zeng, Xiao-Tian Li, Rong-Pei Liu, Shi-Shi Huang, Xiong Zhang, Jian-Yong Wang

**Affiliations:** Department of Neurology, Institute of Geriatric Neurology, the Second Affiliated Hospital and Yuying Children’s Hospital, Wenzhou Medical University, Wenzhou, China

**Keywords:** Parkinson’s disease, freezing of gait, levodopa challenge test, dopamine-induced FOG, case report

## Abstract

**Introduction:**

Freezing of gait (FOG) is a disabling and heterogeneous symptom in patients with Parkinson’s disease (PD). Among them, dopamine-induced FOG is rare and difficult to identify. The treatment of dopamine-induced FOG is complex.

**Case presentation:**

We herein presented a case of PD patient who complicated with refractory FOG. It was identified as dopamine-induced FOG during levodopa challenge test. Her symptoms were alleviated after we reduced the total equivalent dosage of levodopa.

**Conclusion:**

Our report emphasizes the importance of levodopa challenge test in identifying different types of FOG, which is very important for further adjusting treatment.

## Introduction

Parkinson’s disease (PD) is a common neurodegenerative disease, with bradykinesia, rigidity, resting tremor, and gait impairment as its key motor symptoms (Kalia and Lang, 2015). The gait abnormalities of PD patients are mainly manifested in speed, stride, arm swing, frequency, asymmetry and variability, which greatly affect their quality of life (Mirelman et al., 2019). Freezing of gait (FOG) is one of the most disabling symptoms in PD patients.

FOG is characterized by episodic inability or marked reduction of progression of effective stepping forward, which typically occur during gait initiation, turning or passing through narrow spaces while walking (Nonnekes et al., 2015). FOG is recognized as one of the main risk factors for recurrent falls in PD patients. Clinically, FOG is usually divided into three subtypes, including dopamine-responsive FOG, dopamine-resistant FOG, and dopamine-induced FOG (Nonnekes et al., 2015).

Dopamine-induced FOG occurs during dopaminergic drug treatment, which is rare and complicating the treatment (Espay et al., 2012). Accurate identification of FOG type and adjustment of treatment will benefit patients.

Herein, we report a female PD patient complicated with dopamine-induced FOG, which was identified during levodopa challenge test. Her symptoms were alleviated after adjusting the medication.

## Case presentation

The patient was a 77-year-old Chinese woman who has been displaying bradykinesia and resting tremor in her right limbs since she was 58 years old. Her medical history included hypertension and diabetes. The patient was diagnosed as PD and treated with madopar (levodopa/benserazide) 100/25 mg three times daily 19 years ago. Her symptoms were relieved and she did not suffer any motor fluctuation. The drug was adjusted to sinemet (levodopa/carbidopa) 200/50 mg three times daily and pramipexole 0.25 mg three times daily 9 years ago because she had difficulty turning over and walking, which only slightly improved her symptoms. The patient experienced repeated falls 3 years ago, even though her medications have been increased to sinemet 200/50 mg three times daily, madopar 200/50 mg three times daily and pramipexole 0.25 mg three times daily. At the age of 75, the patient visited the Second Affiliated Hospital of Wenzhou Medical University due to the worsening gait disorder.

**Figure 1:**
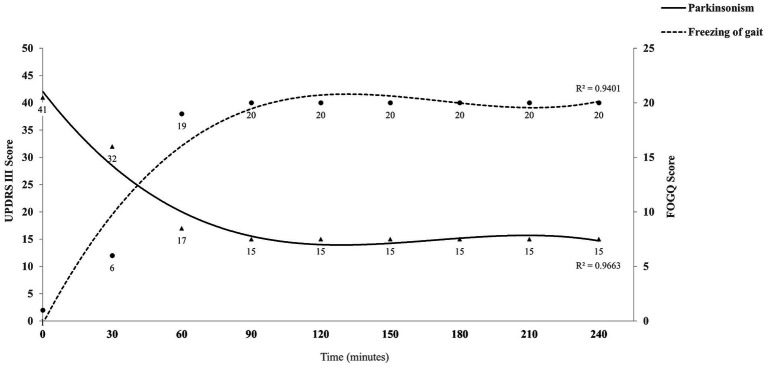
Summary of the levodopa challenge test for the case. UPDRS-III score represents the severity of Parkinsonism (triangular symbols), and FOGQ score indicates the severity of freezing of gait (circular symbols). The cubic polynomial trend line fitted to the data illustrates the overall trend and relationship between variables. UPDRS-III, Unified Parkinson’s disease rating scale part III; FOGQ, Freezing of Gait-Questionnaire.

To investigate the relationship between gait disorder and other symptoms of PD, levodopa challenge test was performed. Interestingly, in the “off” medication state, the patient displayed asymmetric bradykinesia, rigidity and resting tremor (Supplementary Video S1), while her gait was coordinated and smooth (Supplementary Video S2). The unified Parkinson’s disease rating scale part III (UPDRS-III) score at baseline was 41 and the Freezing of Gait-Questionnaire (FOGQ) score was 1. Then, the patient took 300/75 mg of madopar and we evaluated her at 15, 30, 45, and 60 min, and every 30 min thereafter up to 4 h. Her bradykinesia, rigidity and resting tremor were relieved within 45 min, along with the onset of FOG (Supplementary Video S3). Her UPDRS-III score was 17 and FOGQ score was 19 at this point. This phenomenon persisted until the end of the evaluation (Figure 1). We diagnosed the patient with dopamine-induced FOG, and prescribed her with rasagiline 1 mg daily, pramipexole 0.5 mg three times daily, and sinemet 100/25 mg twice daily. We also instruct the patient to refer to internal and external rhythmic cues when walking. The patient’s symptoms including bradykinesia, rigidity, tremor and freezing of gait were alleviated, and the improvement was sustained at 1-month (Supplementary Video S4) and 2-year (Supplementary Video S5) follow-up. It is noteworthy that the patient was diagnosed as PD at the age of 58, and had responded well to low-dose dopaminergic drugs without levodopa-induced dyskinesias for a long time. This suggests that the patient has a rare and benign type of PD. We recommended her to undergo genetic testing, but the patient refused. The patient is satisfied with the treatment.

## Discussion

FOG is a highly debilitating and heterogeneous motor symptom that has been reported in advanced PD, vascular Parkinsonism, progressive supranuclear palsy, multiple system atrophy, corticobasal degeneration, dementia with Lewy bodies, normal pressure hydrocephalus and other neurodegenerative diseases (Factor, 2008). Difficulties in diagnosis and treatment of FOG greatly challenge patients and physicians. In this report, we present a case of female PD patient, whose dopamine-induced FOG was identified during levodopa challenge test. Her symptoms improved after her medications were adjusted.

The mechanisms underlying FOG remain poorly understood. Interactions between different cortical areas, subcortical nuclei and supraspinal regions are thought to be involved in the development of FOG (Gao et al., 2020; Falla et al., 2022). At least four models have been proposed to explain the episodes of FOG, including the threshold model, the interference model, the cognitive model and the decoupling model (Nieuwboer and Giladi, 2013). However, these hypotheses are not sufficient to fully explain the mechanism of FOG.

The prevalence of FOG is directly related to duration of PD, reaching as high as 64.6% in patients with disease duration of more than 9 years (Lamberti et al., 1997; Giladi et al., 2001; Zhang et al., 2021). FOG is highly heterogeneous in clinical manifestations and pharmacology (Schaafsma et al., 2003; Espay et al., 2012; Ehgoetz Martens et al., 2018). Among them, the difference in response of FOG to dopaminergic drugs has received the most attention, and three main types have been identified (Nonnekes et al., 2015). Dopamine-responsive FOG is the most common type. A typical feature is that the FOG occurs in the “off” state and improves together with other motor symptoms when treated with dopaminergic drugs. Dopamine-resistant FOG usually occurs in advanced PD and is often transformed from dopamine-responsive FOG. It responds poorly to dopaminergic drugs, probably due to the involvement of non-dopaminergic mechanisms. Dopamine-induced FOG is a rare and paradoxical phenomenon caused by dopaminergic drugs, which are considered the most effective symptomatic treatment for FOG. As mentioned in our report, patients often increase dopaminergic drugs due to FOG, which in turn worsens the FOG.

The relationship between dopaminergic treatment and FOG in PD is mysterious and complex. Levodopa is the gold standard for symptomatic treatment of PD and is also the first choice for the treatment of FOG (Nonnekes et al., 2015). Meanwhile, previous studies have shown that long-term levodopa treatment is associated with an increased occurrence of FOG (Barbeau, 1971; Jansen et al., 2023). It is believed that co-ordinated motor, cognitive, and limbic circuitry participate in the tight regulation of gait (Lewis and Barker, 2009). Pulsatile long-term levodopa treatment leads to maladaptive plasticity of synapses and increases the mismatch between motor loop on the one hand and cognitive and limbic loops on the other, which further results in the development of FOG. In most cases, the FOG occurs during “off” state because it is more difficult to reach the stimulation threshold of motor loop. While in some rare cases, cognitive and limbic loops are more susceptible to dopaminergic influences, so they are unable to walk despite they feel capable of smooth gait during dopaminergic drug treatment (Nonnekes et al., 2020). The hypothesis needs further investigations.

The treatment of dopamine-induced FOG needs to take both FOG and other parkinsonian symptoms into consideration. Reducing the levodopa dose is a good option. When dopaminergic medication reduction is incompatible due to the unacceptable worsening of other parkinsonian symptoms, subthalamic nucleus deep brain stimulation should be considered (Nonnekes et al., 2015). In addition, the role of visual and auditory cues in improving gait in PD cannot be ignored (Hu et al., 2023). In the case we reported, we reduced the total equivalent dosage of levodopa after determining her FOG type. Fortunately, the dopamine-induced FOG was alleviated, and other PD related dopamine-responsive symptoms remained under control. Here, we propose that for some PD patients with refractory FOG, it is necessary to perform levodopa challenge test to determine whether it is dopamine-induced FOG. Rapid identification of FOG type is very important for adjusting treatment.

In summary, we herein present a rare case of PD patient with dopamine-induced FOG. We emphasize the importance of levodopa challenge test in identifying different types of FOG. Our report adds to the understanding and discrimination of FOG in PD.

## Data Availability

The datasets presented in this article are not readily available because of ethical and privacy restrictions. Requests to access the datasets should be directed to the corresponding authors.
