# Supplementary material for: Case report: Levodopa challenge test is important in identifying dopamine-induced freezing of gait in patient with Parkinson’s disease
Source: Front Hum Neurosci. 2024 Sep 4;18:1464152. doi: 10.3389/fnhum.2024.1464152 (PMC11408169; doi:10.3389/fnhum.2024.1464152)
Supplement: Supplementary file 1 [file Table_1.DOCX]

**Supplementary videos legends**

**Supplementary video 1.** Symptoms evaluation in the baseline. The patient displayed asymmetric bradykinesia, rigidity and resting tremor.

**Supplementary video 2.** Symptoms evaluation in the baseline. The gait of the patient was coordinated and smooth.

**Supplementary video 3.** Symptoms evaluation in the “on” state. Her bradykinesia, rigidity and resting tremor were relieved, along with the onset of FOG.

**Supplementary video 4.** Symptoms evaluation in the 1-month follow-up.

**Supplementary video 5.** Symptoms evaluation in the 2-year follow-up.
